# Human-Mediated Emergence as a Weed and Invasive Radiation in the Wild of the CD Genome Allotetraploid Rice Species (*Oryza*, Poaceae) in the Neotropics

**DOI:** 10.1371/journal.pone.0002613

**Published:** 2008-07-02

**Authors:** Gérard Second, Germinal Rouhan

**Affiliations:** 1 UMR DIAPC, IRD, Montpellier, France; 2 IRD US84, National Museum of Natural History, Paris, France; 3 The New York Botanical Garden, Institute of Systematic Botany, New York, New York, United States of America; Max Planck Institute for Chemical Ecology, Germany

## Abstract

**Background:**

The genus *Oryza* is being used as a model in plant genomic studies although there are several issues still to be resolved regarding the spatio-temporal evolution of this ancient genus. Particularly contentious is whether undated transoceanic natural dispersal or recent human interference has been the principal agent determining its present distribution and differentiation. In this context, we studied the origin and distribution history of the allotetraploid CD rice genome. It is endemic to the Neotropics but the genus is thought to have originated in the Paleotropics, and there is relatively little genetic divergence between some orthologous sequences of the C genome component and their Old World counterparts.

**Methodology/Principal Findings:**

Because of its allotetraploidy, there are several potential pitfalls in trying to date the formation of the CD genome using molecular data and this could lead to erroneous estimates. Therefore, we rather chose to rely on historical evidence to determine whether or not the CD genome was present in the Neotropics before the arrival of Columbus. We searched early collections of herbarium specimens and studied the reports of explorers of the tropical Americas for references to rice. In spite of numerous collectors traveling inland and collecting *Oryza*, plants determined as CD genome species were not observed away from cultivated rice fields until 1869. Various arguments suggest that they only consisted of weedy forms until that time.

**Conclusions/Significance:**

The spatio-temporal distribution of herbarium collections fits a simple biogeographical scenario for the emergence in cultivated rice fields followed by radiation in the wild of the CD genome in the Neotropics during the last four centuries. This probably occurred from species introduced to the Americas by humans and we found no evidence that the CD genome pre-existed in the Old World. We therefore propose a new evolutionary hypothesis for such a recent origin of the CD genome. Moreover, we exemplify how an historical approach can provide potentially important information and help to disentangle the timing of evolutionary events in the history of the *Oryza* genomes.

## Introduction

*Oryza* L. is one of the twelve rice genera recognized in the tribe *Oryzeae* Dumort. of the grass family Poaceae [1], [2]. Conventionally, it consists of 22 wild species which have either diploid (A, B, C, E, F, and G) or allotetraploid (BC, CD, HJ, and HK) genomes defined on the basis of chromosome pairing in interspecific hybrids (genomes A to F) and/or molecular analyses [3], [4], [5]. Its distribution is worldwide in the tropics [6]. The phylogenetically basal *Oryza* sections (F, G, HJ, and HK genomes) have a distribution restricted mainly to Asia and New Guinea in the Paleotropics [4], [6]. Only the phylogenetically most derived *Oryza* section containing the ‘*O. sativa* complex’ (A genome) and the ‘*O. officinalis* complex’ (B, C, E, BC, and CD genomes) has a pantropical distribution.

In the Neotropics (from Southern Mexico to Northern Argentina), only four wild *Oryza* species are found and all are endemics: *O. glumaepatula* Steud. (with the A genome) and *O. alta* Swallen, *O. grandiglumis* (Döll) Prodoehl, and *O. latifolia* Desv. with the allotetraploid CD genome [6]. Two rice species, *O. sativa* L. and *O. glaberrima* Steud., both with the genome A, were independently domesticated thousands years ago in Asia and West Africa, respectively [7], [8]. These cultivated species were introduced into the Neotropics after the European discovery of the New World [9], [10]. West-African slaves were instrumental in developing the crop in the Americas and *O. glaberrima* was probably the first rice grown there [11], [12] where it is not common anymore. Numerous introductions of *O. sativa* from Madagascar, Asia and Africa to the Americas doubtlessly followed [13], [14] and numerous weedy forms commonly found in Africa and Asia, with the A genome but also the C and BC genomes, were probably inadvertently introduced at the same time.

The origin of the CD genome, endemic to tropical America, is puzzling since the C genome is also found both at the diploid and allotetraploid (BC genome) levels in Asia, Papua New-Guinea and Africa [6] whereas the D genome is neither known in other genomic combinations, nor at the diploid level. The E genome, characteristic of the diploid *O. australiensis* Domin from Australia, is the most closely related to the D genome [4], [15]. Therefore, trying to explain the origin of the CD genome necessitates studying and clarifying the spatio-temporal evolution of the genus *Oryza*.

Several hypotheses have been proposed to explain the pantropical distribution of *Oryza*. Until recently, vicariant events during the breakdown of the Gondwanaland super continent have been invoked [3], [16], [17], but this hypothesis is neither supported by fossil data [18] nor by any phylogenetic result [2], [19]. In contrast, G. Second proposed that *Oryza* originated in Eurasia during the Tertiary Era and migrated, by animal-mediated land dispersal alone, to Africa and Australasia [19], [20], [21]. This led the diploid B, C, and E genomes being isolated on the African, Asian and Australasian continents, respectively. These ancestral distributions were later disturbed by human activities after the domestication of rice. The distribution of numerous *Oryza* species, including the four species that are endemic to the Americas (*O. alta*, *O. glumaepatula*, *O. grandiglumis*, and *O. latifolia*) cannot be explained by land migration alone because they are most closely related to taxa occurring on distant landmasses. They are therefore hypothesized to have diverged from wild *Oryza* species after human activities modified their spontaneous distributions and possibly favored their hybridization. Such disturbance accompanied the successful introduction of cultivated rice into the Neotropics.

The introduction of wild *Oryza* species may have resulted, first, from the inadvertent transport of seeds of wild rice as contaminant of cultivated rice. Alternatively, seeds may have remained with the straw (from cultivated or wild rice) that was used during the transportation of animals and for making mats that were largely used on ships, as documented in the case of Portuguese navigators (J. Carney, pers. com) [22], [23]. The introduction of some wild *Oryza* species may also have resulted from the deliberate transport of their seeds by sailors as curios since there was a great interest in exotic flora at the time of the Great Explorations during the 17^th^ and 18^th^ centuries.

The scenario of land-migration is testable because it is constrained by the paleoenvironmental conditions in relation to the specific ecological requirements of the *Oryza* species. The variations of climate, ocean level, orogeny (in particular the rise of the Himalaya), and tectonic movements (in particular those of Australasia relative to islands of Southeastern Asia), make successful land-migration of *Oryza* species possible only at some epochs. Most parsimonious, yet testable, hypotheses should be considered most likely until they can be rejected, but this simple land-migration scenario has neither been thoroughly considered nor accepted. Nevertheless, Vaughan *et al.* [18], [24] suggested that “the movements of animals, including humans and birds” could explain the biogeographical distribution of the *Oryza* species. However, they suggested a large contribution of natural transoceanic dispersal (TOD), for example between Sri Lanka and Africa, and between the Paleotropics and the Neotropics. We do not reject TOD *a priori*, but a combination of several extremely rare events would be needed to explain the origin of the CD genome by hybridization of genomes that are currently found on different continents. TOD therefore does not provide a parsimonious evolutionary scenario and could only be favored if some data (*e.g.* paleontological, historical, or phylogenetic) excluded the possibility of a major human influence in the introduction of *Oryza* to the Americas.

**Figure 1:**
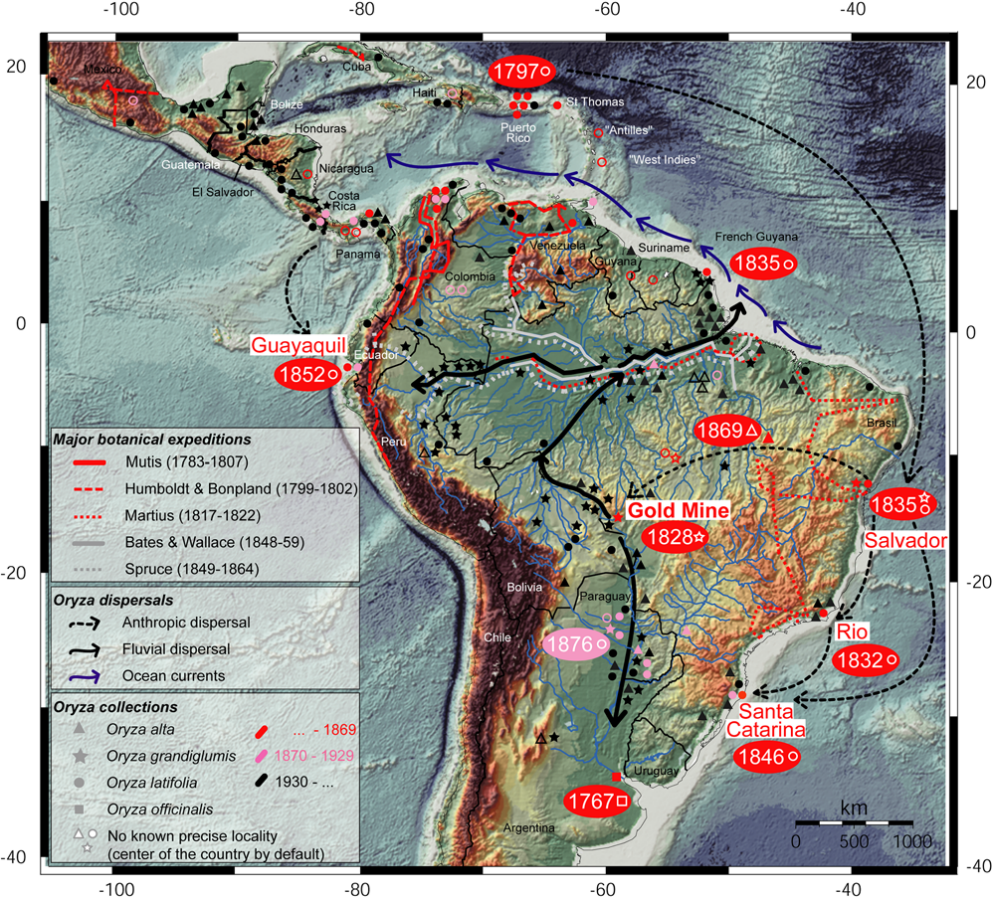
Distribution map of herbarium specimens in relation to time of collection and explorers routes. Summary of the original localization of all specimens determined as belonging to the ‘*O. officinalis* complex’ recorded in our inventory of specimens from the 18^th^ century. All are thought to represent the CD genome, with the single exception of one specimen of *O. officinalis* (C genome). Three epochs are distinguished: 1/ until 1869, 2/ from 1870 to 1929, and 3/ after 1930. The three species with the CD genome (*O. alta*, *O. grandiglumis*, and *O. latifolia*) are distinguished, and the routes followed by major explorers who made collections or mentioned *Oryza* in their reports before 1869 are indicated. Also, key dates are reported for first collections at important sites or in major areas.

Several historical observations support an anthropogenic post-Columbian introduction of *Oryza* to the Americas. Firstly, Amerindians are known to have regularly collected the two other big-seeded genera of the tribe *Oryzeae* that are present in the Americas, *i.e. Zizania spp*. in temperate North America [14], [25], [26] and *Rhynchoryza subulata* (Nees) Baill. in temperate and subtropical areas of South America [27] but there is no record of any substantial interest of Amerindians in *Oryza* which is presently more widely distributed. Secondly, the Amerindian languages do not have any specific names for rice; some of them use a word meaning “water corn”, which is also used for *Rhynchoryza subulata* [10]. Thirdly, in contrast to the situation in other continents, the habitat of *Oryza* in the Neotropics is restricted to disturbed areas (*e.g.* river banks), although large undisturbed areas presumably suited for the growth of wild *Oryza* species are available (G.S., pers. obs.; Fig. 1).

Therefore, post-Columbian human activities appear as the most parsimonious hypothesis to explain the presence of *Oryza* in the Neotropics and this paper aims to test this hypothesis. To do this, we have examined the available evidence for the recent emergence of CD genome species from wild species introduced by humans in the Americas. While a human introduction of wild species of rice from the Old World is clearly plausible, that of *O. australiensis* from Australia requires a specific explanation since historical human exchanges between Australia and the Americas are less obvious. The most parsimonious hypothesis is that *O. australiensis*, which was used as a main source of grains by the Australian aborigines [28], [29], attracted the interest of European navigators and was introduced as a curio in a cultivated rice field of the Neotropics. The first direct contacts of Europeans with Australian aborigines were unequivocally established to have happened in 1606 but could also have occurred slightly earlier [30], [31]. The early 17^th^ century would therefore represent the earliest possible time for the emergence of the CD genome in the Americas.

The *Oryza* genomes are currently being intensively examined [32], providing important information to decipher the evolution of species and genomes. It may seem straightforward to use a molecular dating method to either accept or reject the possibility of a recent human influence in the origin of the allopolyploid CD genome. However, in the case of an allopolyploid, the DNA sequences to be compared to those of the parental diploid species have evolved in the same genomic background with potential consequences that are not completely understood yet [33]–[38]. Therefore, at this stage, we propose that the historical documentation may be the most appropriate source of information to determine the timing of events in the evolution of the *Oryza* CD genome.

## Methods

The occurrence of *Oryza* species and the evolution of their distribution areas in the Americas were inferred from herbarium specimens and the relevant literature including ancient floras and explorers' reports. The itineraries of the major botanical expeditions were plotted onto a map to compare itineraries and locations of the *Oryza* collections (Fig. 1). Ancient herbarium specimens collected before 1899 were examined as exhaustively as possible in the following 23 herbaria, mostly from the Western Old World (abbreviated acronyms according to Holmgren *et al.* [39]): Angers (ANG), Bruxelles (BR), Cambridge (CGE), Chicago (F), Copenhagen (C), Edinburgh (E), Geneva (G), Karlsruhe (KR), Leiden (L), Le Mans (LEMA), Lyon (LY), Madrid (MA), Montpellier (MPU), Moscow (MHA), Munich (M), Nantes (NTM), New York (NY), Oxford (OXF), Paris (P), Saint Petersburg (LE, WIR), Stockholm (S), and Utrecht (U). The distribution of *Oryza* in the 20^th^ century was deduced from surveys by Prodoehl [40], Chevalier [41], and mostly Vaughan [6] who examined and annotated numerous specimens in 14 herbaria worldwide. Thus, for the 20^th^ century, specimens were not exhaustively inventoried here, but online databases (from herbaria in CAY <database ‘Aublet’>, MBG <database ‘Tropicos’>, NY, US, and also from the network database Gbif), along with the associated literature, were used to check that no large areas had been overlooked in determining the current distribution of the genus. Digital images of all examined herbarium specimens collected before 1899 were obtained (Tables 1; Table S1) and are available upon request.

**Table 1:** Specimens of the ‘*O. officinalis* complex’, collected in the Americas in the 18^th^ and 19^th^ centuries, as observed from herbariums or as referenced from literature.

Species	Country a	Locality	Collector's name	Date of collection a	Herbarium b	Name of image (images available upon request), or reference
*O. officinalis*	Argentina	Buenos Aires	Commerson	1767	NTM	NTM1
*O. latifolia*	Puerto Rico	–	A.P. Ledru	1797	E, MPU, P	E1, MPU1, P1
*O. latifolia*	Puerto Rico	–	A.P. Ledru	1806	G	G6
*O. latifolia*	Puerto Rico	–	E.P. Ventenat	<1808	G, P	G9, G10, P5 (type)
*O. latifolia*	Puerto Rico	–	–	<1813	P	P15
*O. latifolia*	Virgin Island	S^t^ Thomas	A. Riedlé	1796–1798	MPU, P	MPU3, P6
*O. latifolia*	Puerto Rico	–	A. Riedlé	1775–1801	MPU	MPU2
*O. latifolia*	Caribbean	–	O.P. Swartz	1783–1786	G	G7, G8
*O. latifolia*	Colombia	Río Magdalena	J.C.B. Mutis	1783–1808	MA	MA1, MA3
*O. latifolia*	Colombia	Río Magdalena, Garrapata	A. Bonpland	1801	P	P2
*O. latifolia*	“Antilles?”	–	A. Bonpland	1801	P	P3
*O. latifolia*	Venezuela	“Venezolanishes Guyana”	A. Humboldt	1801		[40]
*O. latifolia*	Puerto Rico	–	P. de Beauvois	<1820	G	G1
*O. latifolia*	Panama	Porto Bello	J. I. Billberg	1826	S	S2
*O. latifolia*	Brazil	Rio de Janeiro	L. Riedel	1832	LE	WIR3, WIR4
*O. latifolia*	Virgin Island	S^t^ Thomas	–	<1833	P	P4, P8
*O. latifolia*	French Guyana	Cayenne	F.R. Leprieur	1835	G, L	G3, L16, longer glumes
*O. latifolia*	Brazil	Valle Broco	B. Luschnath	1835	LE	LE2, LE3
*O. latifolia*	Brazil	–	B. Luschnath	1831–1837	LE	LE1
*O. latifolia*	Surinam	–	F.L. Splitgerber	1838	L	L13, L17
*O. latifolia*	Surinam/Guyana	–	W.R. Hostmann	1843	G, U	G4, G5, U1
*O. latifolia*	Brazil	Santa Catarina	C. Pabst	1846–1852		[40]
*O. latifolia*	Panama	Chagres	A. Fondler	1850	K	[6]
*O. latifolia*	Ecuador	Puná Island	N.J. Anderson	1852	S, US	S1, [6]
*O. latifolia*	Colombia	–	J.H. Holton	1852	NY	[41]
*O. latifolia*	Panama	–	S. Hayes	1860s	BM	BM5, BM6, BM7
*O. latifolia*	Nicaragua	Grenada	P. Leroy	1869	P	[41]
*O. latifolia*	Colombia	–	E. Andre	1875	K	[6]
*O. latifolia*	Paraguay	San Salvador	B. Balansa	1876	BR, K, L, P, US	BR2, L14, P11, P12, [6]
*O. latifolia*	Trinidad	–	K. Finlay	1877	GH, K	[6]
*O. latifolia*	Paraguay	Cordillere del Mbatobi	B. Balansa	1888	L, P	L15, [6]
*O. latifolia*	Paraguay	–	T. Morong	1888–1890	BM, K, US	[6]
*O. latifolia*	Ecuador	Puná island	H.F.A. Eggers	1892	LE	LE11
*O. latifolia*	Costa Rica	Puerto Viejo	P. Bioley	1893	US	[6]
*O. latifolia*	Costa Rica	Shirores forests	A. Conduz	1895	US	[6]
*O. latifolia*	Costa Rica	Río Diquis	H. Pittier	1897	US	[6]
*O. latifolia*	Colombia	Magdalena	A.C. Smith	1899	L	[6]
*O. latifolia*	Colombia	Santa Martha	H. Smith	19^th^	MPU	MPU8
*O. latifolia*	Brazil	Amazon, Pará (e)	E.A. Goeldi	1884–1907	NY	[41]
*O. latifolia*	Mexico	Laguna de Peralta	J.N. Rovirosa	late 19^th^	NY	[41], note: longer glumes
*O. latifolia*	Haiti	Bayeux	G.V. Nash	late 19^th^	NY	[41]
*O. latifolia*	Paraguay	Río Pilcomayo	Rojas	late 19^th^		[40]
*O. alta*	Brazil	Tocantins	W.J. Burchell	1869	BR, E	BR1, E2
*O. alta*	Paraguay	Rio Paraguay	B. Balansa	1874	L	L1
*O. alta*	Brazil	–	L. Riedel	1880	K	[6], [41]
*O. grandiglumis*	Brazil	Río Guaporé	L. Riedel	1828	K, WIR	K, WIR1, WIR2 (types)
*O. grandiglumis*	Brazil	–	L. Riedel	1828	P	P9, P10
*O. grandiglumis*	Brazil	Valle Broco	B. Luschnath	1835	LE	LE4
*O. grandiglumis*	Paraguay	Pilcomayo River	T. Morong	1889	NY	[41]

a As indicated on the label, or as deduced from the locality and/or literatures, and explorer's reports.

b Herbarium acronyms, according to Holmgren *et al.* [31].

## Results

### Herbarium Specimens

The oldest *Oryza* herbarium specimen from America was found in the herbarium of Nantes (France). It was collected in 1767 in Argentina (NTM1, Table 1) by Commerson. The collector identity and collection date were respectively deduced from Commerson's handwriting (as in P and NTM) and year of his trip to Argentina. The specimen was unambiguously identified as the diploid *O. officinalis* Wall. ex G. Watt (C genome) species, based on the narrow leaves and basal panicle branches whorled with spikelets inserted half way or more from base [6].

The next oldest *Oryza* specimens were collected in 1797 by Ledru in Puerto Rico and also in the Virgin Island formerly known as S^t^ Thomas during the same trip [42]. These were found in several duplicates, some clearly labeled and other likely duplicates in the herbaria of Paris, Montpellier, Geneva, and Edinburgh (Table 1). They are without doubt specimens of *O. latifolia*. Many additional specimens of *O. latifolia* were collected by at least 31 different collectors up to the end of the 19^th^ century (Table 1). Before 1876, these collections all originated from the circum-Caribbean region, including Central America and the Northern coast of South America down to French Guyana (Fig. 1), with four exceptions corresponding to locations near major harbors (Rio de Janeiro, San Salvador da Bahia and Santa Catarina in Brazil, and Puná Island near Guyaquil in Ecuador). All specimens were morphologically similar and corresponded well with the general description of *O. latifolia* but with three exceptions. The first one is a specimen from French Guyana (G3 and L16, Table 1), collected in 1835 which seems to fit the description of the specimen “Rovirosa 314” from Mexico, housed in herbarium NY as reported by Chevalier [41]. It could be related to *O. grandiglumis* because the spikelets are much longer (8 mm) than in *O. latifolia* specimens collected before 1900. However, the sterile lemmas are only half the length of spikelets, linear and scarious, whereas sterile lemmas are wide, coriaceous and the same length as the spikelets in *O. grandiglumis*. A second anomalous specimen was in a collection made in the 1860s in Panama (BM5, 6 and 7, Table 1) which includes several plants, some of which are awnless and therefore do not fit the common description of *O. latifolia*. A third *O. latifolia* specimen (LE2 and LE3, Table 1), together with an *O. grandiglumis* specimen (LE4, Table 1) were collected near Salvador da Bahia by the same collector (Luschnath), both in the same year (1835) and in the same place. This place was called “Valle Broco”, which means a “valley with a nascent stream” that is therefore a suitable place to grow rice (J. Prado, pers. com.). We are unaware of any wild *Oryza* specimen collected from the Salvador da Bahia area since then, and we therefore suspect that these specimens were probably collected from a cultivated rice-growing area. Both specimens appear to be from more vigorous plants and to have longer spikelets than the contemporaneous or earlier specimens of *O. latifolia* collected in the Caribbean area. However, the length of their spikelets do not reach the described limit given for *O. alta* (spikelets >7 mm long [6]). No mention of the exact ecological habitat was found on any of the observed *O. latifolia* specimens, with the exception of one collected in a cultivated area in Surinam (L17, Table 1).

Humboldt and Bonpland, although traveling through vast areas of South America between 1799 and 1802, collected only two specimens of *O. latifolia* in 1801. One collection was made in “Venezuelan Guyana” (Venezuela). This specimen was in Herbarium Willdenow, # 17526, and was probably lost but is mentioned by Prodoehl [40]. Another collection was made in the Río Magdalena area of Colombia. Similarly, the Spanish explorer Mutis [43], [44] collected *O. latifolia* during his expedition (1783–1807) along the Río Magdalena (MA1 and MA3, Table 1). This river was a point of entry from the Caribbean area to Colombia for Spanish colonizers (the city of Mompox, on its banks, is reputed to have been founded in 1537; www.nationmaster.com/encyclopedia/Santa-Cruz-de-Mompox). However, no additional specimens could be retrieved from the historical American collections in the herbarium of Madrid.

The first CD genome specimen clearly derived from the inland Neotropics is the type specimen of *O. grandiglumis* in WIR (WIR1 and WIR2, Table 1), that was collected in 1828 by Riedel near the geodesic centre of South America along the Río Guaporé in Brazil, near the foundation site of Cuiaba city. Duplicates of this collection are present in K and P but the exact origin (Río Guaporé) is only mentioned on the labels in WIR and K. It is morphologically similar to the *O. grandiglumis* mentioned above (LE4) that was collected near Salvador da Bahia seven years later. The first specimen of *O. grandiglumis* away from any area under human influence was collected in 1889 along the Pilcomayo River (Table 1), and the first authentic *O. alta* specimens were collected inland in the Tocantins State of Brazil in 1869 (BR1, Table 1), then in 1874 along the Paraguay River (L1, Table 1).

In addition to the oldest specimens recorded in Table 1, Fig. 1 highlights more recent specimens collected during the late 19^th^ century and throughout the 20^th^ century (pink and black dots, Fig. 1). Although not exhaustive of all the specimens collected during this period, the locations of the specimens shown on the map are representative of the current distributions of the three wild Neotropical CD genome *Oryza* species. In contrast to *O. latifolia,* which had already been collected from the late 18^th^ century in the Caribbean area (including Mesoamerica on both the Atlantic and the Pacific slopes), *O. grandiglumis* was first reported in inland locations and only much later in the Caribbean: its first occurrence was in 1954 from the coast of French Guyana [6]. This region is known to receive mud (probably including seeds) from the mouth of the Amazon (G.S., pers. obs.). *Oryza grandiglumis* was later observed in 1998 in Costa Rica, also on the Atlantic slope [45], [46]. No earlier collection of *O. grandiglumis* is known neither in Costa Rica (C. Arrieta-Espinoza and A. Zamora, pers. com.), nor anywhere else on the Pacific coast. *Oryza alta* was also collected only after the mid 20^th^ in the circum-Caribbean area and only on the Atlantic slope as shown in Fig. 1. It has not been collected in Costa Rica (but some specimens of *O. glumaepatula* had been misclassified as *O. alta*; A. Zamora, pers. com.).

### Early Floras, Explorers' Reports and Relevant Literature

We present in the chronological order of their inception the various pieces of information which we gathered.

The Portuguese explorer Gabriel Soares de Souza, known for his treatise “Tratado descriptivo do Brasil”, written in 1587 [9], considered that the first introduction of rice in the Neotropics was to the Bahia State of Brazil. With regard to the Spanish explorers, the only reference to *Oryza* in the database of the Royal Botanical Garden of Madrid is to the Mutis expedition (1783–1807) on Río Magdalena, Colombia [43], [47], [48]. A detailed color drawing of *O. latifolia* (MA2, Table S1) is found in the report of his expedition [43]. The routes followed by major expeditions of Mutis, Humboldt and Bonpland, Martius, Bates and Wallace, and Spruce are shown in Fig. 1.

Desvaux described *O. latifolia*, noting that it is quite distinct from all varieties of cultivated rice, and reported its distribution as being in “Carolina and Puerto Rico” [49]. It is the first wild species of *Oryza* clearly described from the Neotropics (*O. perennis* Moench published in 1794, was described from a plant cultivated in a European botanical garden, and is a *nomen dubium* [50]). Aimé Bonpland accompanied Alexander von Humboldt throughout his travels in Mexico and South America which lasted for five years from 1799 onwards [51]. Most of their plant specimens were identified by the German botanist Kunth, who mentions the occurrence of *O. latifolia* on the banks of the Río Magdalena [52], [53].

Trinius [54] considered that there were only two species in the genus *Oryza* which were *O. sativa* and *O. subulata* Nees. The latter is distributed in Southern Brazil, but is now recognized to be a nomenclatural synonym of *Rhynchoryza subulata* rather than an *Oryza* species.

From 1817 to 1820, Martius explored a large part of Brazil [55] and with the collaboration of sixty five additional botanists, produced one of the outstanding botanical works of all time: the monographic flora series *Flora brasiliensis* [56] containing taxonomic treatments of 22.767 species, almost all being Brazilian angiosperms. The *Flora brasiliensis* is the only complete Flora ever published for Brazil (now online at http://florabrasiliensis.cria.org.br/index). In this Flora, Döll [57] described the tribe *Oryzeae* and documented the distribution areas of its constituent genera (that were then recognized in South America). However in *Flora brasiliensis*, specimens that clearly belong to *O. latifolia* and *O. grandiglumis* in their present acceptation (in particular the Luschnath collection from near Salvador da Bahia; L2, L3, L4, Table 1) were treated as varieties of *Oryza sativa* which is described as being distributed in “marshy areas with hot climate, not far from human settlements, mainly from regions of Pará, High-Amazonia, Mato-Grosso, Goiás, and Minas Gerais.” Morphologically intermediate forms were mentioned that now appear to belong to *O. sativa* (C2 and C3, Table S1). No fewer than six collectors (Warming, Luschnath, Martius, Spruce, Riedel, and Burchell) are cited and all precise itineraries and bibliographical references are detailed by Martius in the first part of *Flora brasiliensis* [56]. Later, Döll identified another specimen (BR1, Table 1) as *O. latifolia* Desv. but this specimen is not mentioned in his contribution to *Flora brasiliensis*. *Oryza glumaepatula* is referred to in a note (as its synonymous *O. caudata* Trin. ex Döll), as being distributed in Surinam and the States of Pará and of Mato Grosso in Brazil. *Oryza subulata* Nees (now *Rhynchoryza subulata*) is mentioned as being from the State of Río Grande do Sul and from Paraguay and Uruguay. No mention is thus made of the occurrence of the genus *Oryza* along the Amazon where the four wild species are now locally abundant although these plants would have been easy to identify from a boat. Indeed, traveling along the rivers by boat was the best (and usual) way for explorers to penetrate and explore inland (see Fig. 1). Moreover, the best time to see fertile wild rice is during high water in the beginning of the drier season and is thus also the best time to penetrate inland by boat on inundated plains.

Based on our records, Bates [58] (p. 194), in 1849, was the first to mention the observation of wild rice in the lower Amazon “which Amerindians have never reclaimed, although they have adopted the plant (rice) introduced into the country by Europeans.” During the same period, Gardner [59] wrote about rice cultivation but does not mention any occurrence of wild rice. Spruce (cited by Wallace [60]) collected *O. glumaepatula* (C1, Table S1) along the lower Amazon. In a note written after his return to England, he mentions that he had observed, around 1850, the collection from canoes of wild rice commonly encountered along the tributaries to the Amazon. But none of his specimens represent a CD genome species (see [40] for more details). Wallace traveled in Amazonia from 1848 to 1852 [60], [61], partly with Bates and Spruce. His herbarium specimens and most of his notes were lost while returning to England because of the destruction of his ship by fire. It is interesting to note what he wrote after visiting a steam-driven rice mill in the Pará State about “The Pará Rice” [61]: “No care is taken in choosing seeds or in preparing ground; (... and rice...) is therefore seldom cultivated on a large scale, the greater portion being the production of Indians and small landholders, who bring it to the mill to sell.”

German botanists were very active in South America in the 19^th^ century, but most of their herbarium collections (including that of Martius) were lost during World War II. Nevertheless, due to their collaborations worldwide, some of their *Oryza* specimens were sent to other herbaria (mostly LE and WIR) where they still exist and can be examined. Moreover, we have the account of Prodoehl [40] of *Oryza* species which had been collected. Again, in the German collection, the earliest *O. latifolia* specimens were collected exclusively in the Caribbean area, and only later (after the mid-19^th^) from Southern Brazil and Paraguay. However, Prodoehl mentions a single specimen of *O. grandiglumis* “Riedel 1261” which is the type specimen that was collected inland, in April 1828, along the Río Guaporé (WIR1, Table 1). This river is a tributary of the Amazon River, and has its source (as also the Paraguay River) near a major gold mine which is the foundation site of Cuiaba city (Fig. 1).

Chevalier [41] mainly studied collections in the herbaria P and NY. He considered *O. latifolia* to be a very variable species (in contrast to its lack of diversity that we noted in early specimens from the Caribbean area). Portères [62] interestingly documented the distribution of *O. latifolia* as extending to “nearly all Caribbean islands” and described this species with several varieties. Based mostly on the collections from the 20^th^ century, Vaughan [6] recognized the three South American species of the ‘*O. officinalis* complex’ consisting of *O. alta, O. grandiglumis*, and *O. latifolia*. In *O. latifolia*, he distinguished both a short and a tall form. His records are pointed in Fig 1.

Although not a botanical monograph, the work of Cascudo [10] is pertinent to our survey because it includes an attempt to a review of all information concerning the question of whether or not the genus *Oryza* was present in Brazil prior to 1500. He came to the conclusion that there was no *Oryza* in Brazil prior to the arrival of Europeans. He cited the poet Santa Rita Durão who, in his famous *Caramaru* written in 1778–81, mentioned the presence of “wild rice” in Cuiabá and the Pará State. Cascudo also cited Couto de Magalhães who had recorded that, in about 1863, nomadic Guato Indians had been observed collecting wild rice in the Pantanal (frequently inundated) near Cuiabá. However, he also noted that this observation was made three centuries after the arrival of Europeans. He suggested that the undisputed occurrence of *Rhynchoryza subulata* in this region before their arrival may have been responsible for the controversies (see [63]) about the existence of *Oryza* in Brazil before 1500. He stated that rice was not part of the diet of the indigenous people before its introduction by Europeans, particularly to North Brazil. Cascudo specified that rice (“*da terra*”) had been first cultivated in Brazil in the Bahia State, and he added (p. 517) that this rice was red and dehulled by mortar and pestle. This suggests that the varieties cultivated around 1896–1897 were still, at least in part of the African rice *O. glaberrima.* The presence of this species in El Salvador and French Guyana was also attested by Portères [62], [64] and by two herbarium specimens (G12 and BM8, Table S1). Earlier, the French explorer Auguste de Saint-Hilaire also reported that two types of cultivated rice were distinguished in Brazil: the red rice (“*arroz vermelho da terra*”) and the white rice [65].

## Discussion

### On the Post-Columbian Introduction of *Oryza*

Herbarium specimens, early floras and explorers' reports indicate that European botanists and explorers were interested in American *Oryza* taxa as early as in the late 18^th^ century. The two sites (Puerto Rico and Río Guaporé) of the two type specimens of *O. latifolia* and *O. grandiglumis*, respectively, were subjected to extensive collections still preserved in herbaria, indicating they had received a particular attention at that time. No fact allows rejecting the parsimonious hypothesis of a post-Columbian introduction of the genus *Oryza* in the Americas: there is no explorer report or any herbarium specimen attesting the presence of wild *Oryza* species far from the sites of Europeans activity, and no documentation reports any traditional interest of Amerindians in *Oryza*. In contrast, the presence of *Rhynchoryza subulata* in South America was reported since the early 19^th^ century, and this species is clearly ancient on the continent. Although it displays a much more restricted distribution than contemporaneous *Oryza,* this monotypic endemic genus was collected early, and its use by Amerindians was mentioned by anthropologists [27]. Similarly, in the major *Flora brasiliensis* [56], [57], the *Oryzeae* other than *Oryza* (including in particular *Rhynchoryza subulata*) were precisely treated, with distributions clearly determined in the wild, whereas the distribution of *Oryza* was defined as restricted to “human habitations” and their vicinities.

The earliest mention of “wild rice” found was in a poem [10] from the late 18^th^ century. It indicates two sites (the Pará State, and city of Cuiabá in the Mato Grosso State), which are closely related to early activity of Europeans and associated African slaves in Brazil. However, “wild rice” in this context does not necessarily point to any of the three allotetraploid *Oryza* species considered in the present study. In the Pará State, our survey suggests that the earliest collection of a CD genome species occurred as late as the early 20^th^ century [6] whereas the earliest herbarium specimen of *O. glumaepatula* (A genome) dates back to 1828 (LE5, Table S1). Therefore, the citation of the poet more likely refers to *O. glumaepatula*. The same is probably true for the “wild rice” observed by Bates, Spruce and Magalhães in the Amazon Basin around the mid 19^th^ century. The first *O. glumaepatula* specimen (LE5) bears a clear indication of the species distribution: the Pará and Mato Grosso States, which were also mentioned in both the poem and in *Flora brasiliensis* for the distribution of *O. glumaepatula* (including its synonymous *O. caudata*). This specimen was collected by Riedel in the same year and same place as the first specimen of *O. grandiglumis* collected inland. All this suggests also a limited distribution of *Oryza* at that time.

An important limitation of herbarium-based studies of species distribution (due to contingencies of botanical collections and all risks inherent to their conservation) is that absence of herbarium collection in a locality and during a given period cannot be interpreted as the absence of a species in this locality at that time. Plants like *Oryza* that are easy to locate and collect when fertile, particularly when traveling by boat as was the case of most early explorers. As a consequence, two main factors could explain their absence in herbarium collections of early exploration of the Amazon and Paraguay River basins if one assumes they are native in the Neotropics: early collectors may have not collected *Oryza* either by lack of interest in grasses in general, or because these species were not fertile at the time of the expeditions.

However, most (if not all) early explorers were naturalists with broad and multiple interests, who used to collect extensively (not only plants, but also *e.g.* animals and minerals) and did not focus on any particular groups of plants. Moreover, herbarium data demonstrate a large interest of early collectors for wild and native grasses, including other taxa of the tribe *Oryzeae* that share habitat preferences with *Oryza*. Even if only considering type specimens (thus possibly overestimating the date of first collection), we detected numerous specimens from South America dating back to the late 18^th^/early 19^th^ centuries (following date intervals are given based on the documented time of explorations). These collections thus clearly predate the first inland *Oryza* collections away from cultivated rice fields (first one in 1869, see the Results section), and show that early explorers used to collect grasses as well as any other plants. At the genus level, for example, *Luziola* Juss. was collected in Peru by J. Dombey in 1778–1784, Ruiz and Pavón in Peru or Chile in 1778–1788, Mutis in Columbia in 1783–1808, G.H. von Langsdorff in Brazil in 1813–1830, St Hilaire (unknown location) in 1816–1822, and Spruce in the Brazilian State of Pará in 1849–1864. It is interesting to note that Mutis also collected *O. latifolia* but only in the Caribbean area (Table 1) and St Hilaire described with some details rice culture but did not mention any wild *Oryza* forms [65], Another genus, *Leersia* Sol. ex Sw., was collected in Jamaica by O.P. Swartz as early as 1783 and in Mexico by C.J.W. Schiede in 1836. Third, *Rhynchoryza subulata* (Nees) Baill. was collected by F. Sellow in Paraguay in 1815–1829. Its distribution is given by Kunth [52] as “*regni Paraguayani et in Rio Grande do Sul, locis paludosis*”. The relatively small distribution of this native species was thus fairly well circumscribed from the beginning of the 19^th^ century. Nees von Esenbeck [66] treated it as native, noting that the same was not obvious for *O. sativa*. Last, the genus *Zizianopsis* Döll & Asch. was collected by F. Sellow in Brazil in 1815–1829.

Another reason for the absence of herbarium specimens may be that expeditions did not take place during the *Oryza* flowering period. Although this cannot be completely ruled out, two lines of evidence lead us not to favour this hypothesis.

First, the phenology of *Oryza* species is not highly seasonal: Vaughan [6] observed that *Oryza latifolia* can be collected all year round, and O. *alta* and *O. grandiglumis* flower mostly from March to July and from September to October. Based on the material in herbarium NY only, the 10 fertile specimens of *O. alta* were collected in January, March, May, August, September and December; and the 13 specimens of *O. grandiglumis* were collected in January, March to August and November.

Second, because *Oryza* typically flowers in the wild at the end of the rainy season and matures at the beginning of the dryer season, this is often an appropriate time to travel. Also, travelling by boat during the high water time allows visiting inundated plains, a typical habitat for these species. But in fact, early explorers were always travelling for several months, and even more frequently for several years, mostly by boats in big rivers basins (*e.g.* the Amazon and Paraguay River basins). Thus, their trips most likely covered all seasons. For example, Humboldt and Bonpland explored South America and Mexico during five years (from 1799 onwards). They travelled and collected along the Orinoco River and the Río Negro, from July 1799 to July 1800 that is one entire year. They did not collect *O. alta* nor *O. grandiglumis* which now occur in this area (Fig. 1), although they collected *O. latifolia* in the Caribbean area (Table 1). This is in accordance with Kunth [52], who stated in 1822 that *O. latifolia* was growing in the Caribbean area, in Columbia and Venezuela, and some of its islands, Another example involves the exploration of Martius and Spix, who spent almost three years (from 1817) exploring the Amazon Basin. Their plant collections cover all seasons every year, but they never collected *Oryza* [56], [67]. In Martius' *Flora brasiliensis* [56], *Oryza* is reported to be found only near human habitations on the basis of the collections of six collectors. Finally, the three naturalists Spruce, Wallace and Bates were dominant figures in the British explorations in Amazonia in the mid-19^th^ century: Wallace worked in the region for four years, Spruce for six years, before moving to the Andes (where the CD genome species were collected later along its route), and Bates for eleven years [68]. Especially, Spruce was a recognized botanist interested in economic botany (especially, he collected *O. glumaepatula* as stated above); although not genuine botanists, Bates and Wallace were also collecting plants and made various references to economic plants in their writings [68]. But none of these explorers collected any CD genome *Oryza* species. In contrast to early and extensive collections, it is noteworthy that much more modest trips, during the 20^th^ century, gave the occasion to repeatedly collect the three wild CD genome *Oryza* species.

Therefore, the absence of fertile material when early explorations took place does not appear as plausible explanation for the absence of *Oryza* specimens in herbarium collections.

The observed spatio-temporal distribution of herbarium specimens representing CD genome species are difficult to explained if their distributions before Columbus were similar to their present ones. On the contrary, the observed patterns can be explained in a surprisingly coherent manner by a post-Columbus introduction and expansion, as exposed thereafter in the section ‘Possible Scenario for the Evolution of the CD Genome’.

An unambiguous result is that, in spite of numerous collectors traveling inland (most often along rivers) and collecting grasses (including *Oryza*), plants belonging to CD genome species might not have been observed away from cultivated rice fields before 1869 in the Tocantins, not before 1889 along the Pilcomayo River, not before the 20^th^ century along the Amazon River, and even later in the Caribbean area. Therefore, could the frequent collections of *O. latifolia* from the Caribbean, since 1797, have been weedy forms escaped from cultivated fields?

### On the Emergence of *O. latifolia* as an Agricultural Weed

It is surprising that the early distribution of *O. latifolia* was restricted to the Caribbean area (including the Caribbean part of Columbia [53]) and Carolina (United States), as reported in the original description of the species [49]. Carolina is indeed a location that does not fit the suitable climatic envelope for *Oryza* in the wild. Given the relatively high morphological diversity displayed by *O. latifolia* nowadays (variations from short statured and stunted to high statured vigorous plants; [6], [41], [62]), it is also surprising to find a single phenotype (short statured) dominating the early collections of *O. latifolia*. This nevertheless makes sense if *O. latifolia* was a purely weedy species until the mid 19^th^: as a weed it may have dispersed rapidly due to the diffusion of agricultural varieties of rice, and it could have reached the main South American harbors from the Caribbean along with seeds of cultivated rice (Fig. 1). The specimen L17 (Table 1) supports this hypothesis as it is mentioned to be “cultivated” on its herbarium label: *O. latifolia* has never been cultivated itself, and this specimen can therefore be best interpreted as the collection of a weed growing in or around fields.

Other observations suggest that some *Oryza* species were introduced in Americas as weeds, and probably had only a transient distribution in the area. In particular, the earliest *Oryza* herbarium specimen found (1767, Argentina) was collected by Commerson, the botanist of the first French circum navigation, who also collected similar specimens from Java island, later in the same journey (P13 and MPU7, Table S1). It does not belong to a CD genome species and is the only specimen, in our data, to represent the Asian *O. officinalis* (C genome) in the Americas. This single occurrence is congruent with the introduction of this species as a transient weed in the Americas. Similar plants may have been present in the Caribbean at the same time, constituting the maternal parent of the CD genome. Although not related to the CD but to the A genome, the same scenario may apply to the African *O. barthii* A.Chev., found only once in the present collections of South America (M2, Table S1: specimen from the Martius herbarium, distributed in Para, Río Negro and Mato Grosso, “habitat quasi sponte”, near cultivated fields). The Neotropical weedy forms of *O. latifolia* (of hybrid origin) were probably locally better adapted than the *Oryza* species introduced directly from the Old World which have now disappeared.

### On the Later Emergence of CD Genome Species in the Wild

We found numerous historical *Oryza* herbarium collections belonging to CD genome species. They mostly originated from the Caribbean area (characterized by early and intense European activity), and consisted of *O. latifolia*. On the contrary, there is a scarcity of specimens of CD genome species collected elsewhere in South America and all of them were collected in locations closely related to European activity (*e.g.* early harbors and gold mine). The first two CD genome specimens collected inland in South America are of species *O. grandiglumis* and *O. alta* (in 1828 and 1869, respectively), and these two species were not collected in the circum-Caribbean area before the mid 20^th^ century.

Several intermediate forms between the three CD species currently recognized have been observed in early, but not in subsequent, collections. These include the two specimens from French Guyana and Mexico with long spikelets and long sterile lemmas, and the two specimens with strong culms and spikelets longer than usual, observed in Salvador (Bahia State, Brazil) in 1835. These intermediate forms suggest that *O. latifolia* progressively differentiated into *O. alta* and *O. grandiglumis* in cultivated fields. Some of these morphological variants might have been introduced to the major gold mining site near the Cuiabá area (Mato Grosso State, Brazil) as contaminant of rice seeds. This site was established since 1718 [69] (p.56) near the Pantanal ecosystem, a large swampy area located on the drainage line between the Paraguay and the Amazon Basins and thus connecting them. In this ecosystem, both *O. grandiglumis* and *O. alta* probably expanded their range thanks to their adaptation to dispersal through flotation of seeds (long sterile lemmas of *O. grandiglumis*) or floating mats (in *O. alta*), respectively [6]. They likely colonized the disturbed banks of the two major rivers Paraguay and Amazon during the 19^th^ and 20^th^ centuries, respectively. From the Amazon mouth, they could migrate to the Caribbean area, possibly not before the second half of the 20^th^ century, via the coastal oceanic surface current fed by the Amazon [70]. The location of the gold mine thus appears as an ideal place to select characters of dispersal through flotation, allowing a rapid dispersal in the area of present distribution of vigorous forms in the wild. The fact that *O. alta* also seems to disperse away from rivers banks, more than *O. grandiglumis* (Fig. 1) is consistent with the fact that it retained the usual characteristics of dispersion along with terrestrial animals –awn and hair– as found in most species of the section *Oryza*. On the contrary, *O. grandiglumis* is often awnless, with no hair, like many cultivated rice varieties. It is found along the Paraguay and Amazon Rivers only, with the exception of Meso-America, along the coast boarded by the Caribbean current. The absence of herbarium collection of *O. grandiglumis* in other large river basins of the Neotropics is also consistent with this scenario (Fig. 1).

A simple scenario for a very recent establishment of the vigorous CD genome plants in the wild, together with the absence of evidence attesting the presence of these CD genomes in the wild before the second half of the 19^th^ century suggests that natural transoceanic long-distance dispersal may not be responsible for the presence of this genome in the Americas. This finding highlights that the relative importance of transoceanic long-distance dispersal *vs.* land migration may have been exaggerated when trying to elucidate the biogeographical history of the genus *Oryza* [24]. The present survey is thus congruent with earlier findings [19], which suggested that land migration alone may explain the natural distribution of the *Oryza* section in the Paleotropics; it also supports the view that wild *Oryza* species can be classified as “spontaneous” and “derived” in relation to human interference since the domestication of rice [20].

### Possible Scenario for the Evolution of the CD Genome

Some recent molecular phylogenetic and genomic results on the two components of the CD genome are difficult to integrate to our scenario of recent evolution, unless the two genomes have very different rates of evolution. Although the C genome of various diploid species is closely related to the C components of the BC and CD genomes –which is consistent with the recent emergence of these *Oryza* allopolyploids– there is considerable divergence between the E and D genome. Furthermore there is also substantial divergence between the D genomes of *O. latifolia*, *O. alta*, and *O.grandiglumis* [4], [71]. Despite this, the phylogenetic topology showing *O. latifolia* as the sister lineage to the clade composed of *O. alta* and *O. grandiglumis* [4], [71] is consistent with the temporal sequence of differentiation inferred from herbarium data. Thus if our scenario for the recent formation of the CD complex is correct, the rate of molecular change in the E/D genomic components of CD genome must have been very high. A comparison of the relative size (in Mb/1C [72]) of the CD genome (*O. alta*: 866, *O. grandiglumis*: 891, *O. latifolia*: 806) with its two accepted ancestral genomes (C genome, *O. officinalis*: 549, and E genome, *O. australiensis*: 823) shows that allopolyploidization was accompanied by a substantial loss of DNA, if the donor plants were similar in genome size to the contemporary plants. This last point cannot be certain as there has apparently been a recent retrotransposon-driven expansion of the *O. australiensis* genome [73]. These results do nevertheless indicate that the C component is “pivotal” in the CD genome (in the sense used in the *Triticeae* by Zhohary and Feldman [74]) and that the D genome component is much more plastic and may have experienced a stronger genome shock in the sense proposed by McIntosh [75], This shock would result from the hybridization between species that had been separated for a long time, and which had undergone independent evolution, on different continents. The possibility of rapid elimination of DNA during allopolyploidization was previously demonstrated in cereals *e.g*. in the *Triticeae* [76], [77], [78]. Complex genomic rearrangements have also been described in the wheat hexapolyploid genome [79] and the possibility of different rates of evolution between subgenomes of tetraploid wheat has also been considered [80]. A broader review [34] indicates that, both for *Brassica* and the cereals, rapid genomic changes can occur in newly synthesized allopolyploids and that the nuclear genome of maternal origin usually experiences less change than the paternal contribution. The same has been confirmed in the tobacco (genus *Nicotiana*) including the losses of some retrotransposon sequences subsequent to the allotetraploid tobacco's formation [81], [82]. This is consistent with our hypothesis for the origin of the CD genome. Also, polyploids are now recognized not to be an evolutionary dead end, but rather the result of a dynamic process of evolution [34]. Positive genetic and epigenetic interactions between genomes in hybrids, particularly allopolyploids, are thought to lead to hybrid vigor [35], which also seems to apply well to the present *Oryza* study case. We thus propose, as detailed below, that the D genome never existed at the diploid level but only as a component of the CD genome which represents a product of a diploid-tetraploid complex between the B and C genomes: this BC genome complex probably emerged in the Old World many years before the Australian E genome could integrate it. This integration must have occurred after the domestication process when human exchanges of varieties of cultivated rice (and associated weeds) were intense between Asia and Africa, strongly disturbing the natural habitat of the species.

**Figure 2:**
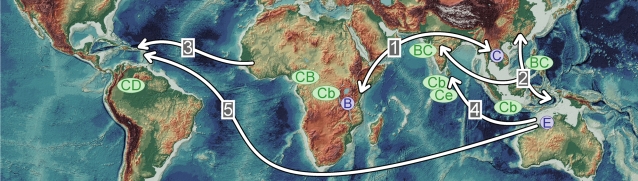
The ‘*O. officinalis* diploid-tetraploid complex’ in relation to major routes of great navigation. Distribution map of the genomes distinguished in the ‘*O. officinalis* complex’, as detailed in Table 2. The three spontaneous diploid genomes (B, C, and E) and the derived genomes or subgenomes are figured in blue and yellow respectively. Arrows indicate presumed routes of human dispersion of *Oryza* across natural geographic barriers exchanges: exchange of goods during many centuries between Asia and Africa (1), and with insular Southeast Asia (2) through navigation; the “Middle Passage” during the triangular trade which involved one way slave trade from Africa to the Americas (3); introduction of *O. australiensis* to Sri Lanka (4) as a curio after the discovery of Australia by Europeans (but possibly earlier); introduction of *O. australiensis* to Caribbean rice fields (5) as a curio during the 17^th^ century from Australia (possibly via Sri Lanka). World map after Calmant *et al.* [87].

While the A genome (as found in cultivated rice) is spontaneously widespread in Africa, Asia and Australasia, the B, C, and E genomes are not. They originated respectively in Africa, Asia and Australasia [19], [20]. Early human-mediated genetic exchanges of *Oryza* species between continents were facilitated by the establishment of navigational routes in the Indian Ocean and to Southeast Asia during (or even before) the Islamic Golden Age (8^th^ to 13^th^ centuries) and by the development of maritime trading networks by the Indians and Chinese centuries before the advent of Columbus or other European explorers [83], [84]. The B and C genomes (of *O. punctata* Kotschy ex Steud. and *O. officinalis* respectively) were thus repeatedly brought into contact. This resulted in two reciprocal allotetraploids being formed [85], [86], from which a diploid-tetraploid complex emerged (Fig. 2; Table 2). The selection of genetic factors (*e.g.* production of unreduced gametes) which favor polyploidization and introgression between different ploidy levels thereby occurred, as proposed for the ‘*Aegilops*-*Triticum* complex’ [88] and also observed in the *Coffea* genus [89]. Unreduced gamete production is known to be under genetic factors determinism; its frequency is observed to be high in certain genotypes although expected to be usually counterselected [36]. Some *Oryza* “Cb” genome forms (C genome introgressed by the B genome, as defined on the basis of a multivariate analysis of isozyme markers) emerged from this complex as is now found in the Southeastern Asian forms of *O. officinalis* often collected around human habitations (the most likely forms to contaminate seeds of *O. sativa* introduced to America) and in the *O. eichingeri* Peter of Africa and Sri-Lanka [19; although controversial]. Hybridization between a C and E genomes in the Paleotropics is also attested by *O. rhizomatis* Vaughan in Sri Lanka (Table 2). This species displays a mitochondrial genome that seems intermediate between those of the C and E genomes [90] and, based on data from nuclear markers, has a closer affinity to *O. eichingeri* collected in Sri Lanka than to *O. eichingeri* collected in Africa [91], [92]. In this context it is also relevant to note that there is a similarity between the sequence of a B genome prolamine gene and its equivalent in the CD genome [93]. This points to a homogenization (concerted evolution) which implies the presence of this B genome prolamine gene in one of the diploid ancestor of the CD genome. Such a rapid homogenization of rDNA genes occurred in *Tragopogon* allotetraploids formed recently (in the last 80 years [94]). We made an attempt to reconstitute (without using embryo rescue) the CD genome by fertilizing a diploid Cb genome plant *O. officinalis* with pollen from the E genome *O. australiensis*. This maternal plant originated from Indonesia and is morphologically similar to the earliest herbarium specimen found in Argentina (NTM1, Table 1) and Java (MPU13 and P13, Table S1). A vigorous perennial triploid F1 hybrid resulted, confirming that the formation of unreduced gametes may not be a rare event in Cb genome plants (G.S., unpubl.). This implies that, in addition to including pivotal and plastic components, the CD genome could be a mosaic between the B, C and E genomes.

**Table 2:** The ‘*O. officinalis* complex’, with a classification of species as ancestral and derived (post-domestication), and the corresponding genomes [see 20].

Species	Distribution	Status	Genome a
*O. punctata*	Africa	Ancestral diploid	B
*O. officinalis*	Asia	Ancestral diploid	C
*O. australiensis*	Australia	Ancestral diploid	E
*O. punctata* (4n)	Africa	Derived tetraploid	CB
*O. minuta*	Philippines, P.N.G.	Derived tetraploids	BC
*O. officinalis* (4n)	India		
*O. alta*	Neotropics	Derived tetraploids	CD
*O. grandiglumis*			
*O. latifolia*			
*O. officinalis*	South East Asia	Derived introgressed diploids	Cb
*O. eichingeri*	Africa, Sri-Lanka		
*O. rhizomatis*	Sri-Lanka	Derived introgressed diploid	Ce

a The first letter indicates the maternal parent of allotetraploids, and the lower case letter indicates which diploid genome was introgressed.

The Dutch may be at the origin of the dispersion of both the E genome from Australia and the Cb donor, facilitating the formation of the CD genome in the Neotropics. They had a large maritime empire during the 17^th^ and 18^th^ centuries (the Dutch Golden Age; [95], [96]), that encompassed all the putative donor and receptor sites. This included Surinam, part of the Brazilian coast north of Salvador da Bahia (this capital itself having been under Dutch control during the year 1624), and especially S^t^ Thomas Island close to Puerto Rico in the Caribbean, that is the area where the numerous earliest *O. latifolia* specimens were collected *ca* 1797. Sri-Lanka and Indonesia (including West Papua) were also part of this empire, and Dutch were the first known Europeans to contact the Australian aborigines in 1606 [31]. Evolutions of *O. rhizomatis* and of the CD genome were not necessarily directly connected to one another and could have been the result of separate hybridization events in the diploid-tetraploid complex. Furthermore there were earlier contacts between Australasia and Asia (for thousands of years [97]) that could have resulted in the C and the E genomes being brought into contact. But there is no evidence of any CD genome that could have been carried to the Neotropics.

Differentiation of *O. alta* and *O. grandiglumis* from *O. latifolia* involved, in addition to the acquisition of high vigor, two morphological features which are otherwise only found in A genome species: long spikelets (found in several species including cultivated rice) and long sterile lemmas (only observed in some varieties of the cultivated species *O. sativa* and *O. glaberrima;* the occurrence of a variety of *O. glaberrima* with long sterile lemmas in El Salvador was reported [62]). As a consequence, we conclude that introgression of genes from cultivated rice into the CD genome probably occurred. This is plausible since the reciprocal introgression of the CD genome into *O. sativa* has been achieved in laboratories [98], [99]. Furthermore, the spontaneous formation of a sterile triploid hybrid (widely dispersed through vegetative means) between *O. latifolia* (probably maternal parent) and *O. glumaepetala* has been described [46]. Non-shattering *O. latifolia* has also been observed (A. Zamora, pers. com.). This, together with the observation that spikelets of *O. grandiglumis* are commonly awnless and airless, two characters commonly associated only in *O. glaberrima*, are in support of our hypothesis. The CD genome could thus be a genome that has evolved enough plasticity to also integrate some components of the A genome, particularly from cultivated rice. If this is correct, the CD genome would include components of the four diploid genomes comprising the section *Oryza* of genus *Oryza* (A, B, C and E); the C genome, for which the Asian origin and the ecological adaptation to wet forests suggest its most ancient origin among the section *Oryza* [19] appears to be the pivotal genome.

In the Neotropics, several factors may have favored introgression in the 17^th^ and 18^th^ centuries. Wallace, after his visit to a steam-driven rice mill, described how rice was cultivated by many people living in that region in precarious conditions. Moreover, many escaped slaves (‘Maroons’) were involved in clandestine rice culture from the late 17^th^ century onwards, as documented by Carney [100]. Consequently, fields were often abandoned, allowing weeds to flourish alongside the crop plants and providing the conditions for interspecific hybridization to occur. This is consistent with the observation from herbarium data that there was a progressive appearance of forms with long sterile glumes and spikelets in fields of cultivated rice. Such introgressive hybridization, in a sympatric zone of exceptional occurrence, between *Coffea arabica* and one of its diploid ancestors has been documented by Mahé [89].

### Conclusion

Our understanding of the genomic mechanisms underlying the possible rapid differentiation of the allopolyploid CD genome is still far from complete. Several factors could have contributed to a greatly accelerated rate of DNA sequence variation in the paternal D genome compared to the maternal C genome. This makes very difficult to date the formation and the diversification of the CD complex by molecular dating methods. We have therefore chosen to consider this question by examining historical herbarium collections and documentation that we interpreted in the light of various phylogenetic, cytologic and genomic results available in the literature. We propose that the emergence of the Neotropical CD genome species occurred recently as a result of human interference rather than before Columbus, as a consequence of long distance natural transoceanic dispersal. The statement of Voeks [101] that “much of the biotic similarity exhibited between Africa and South America can be attributed to 500 years of human-mediated plant and animal dispersion and colonization, yet a complete omission of humans as biogeographical factors is often noticeable in biologists' view of biogeography” would seem to be relevant here also. The CD genome species would result initially from the pollination of weedy forms of *O. officinalis* (Cb genome) or tetraploid *O. punctata* (BC genome) by *O. australiensis* (E. genome). Its diversification and expansion from cultivated fields to inland wild regions would have involved subsequently introgression from pollen of cultivated rice (*O. glaberrima* and/or *O. sativa*).

Some ways to test this scenario include pursuing of historical documentation, studying herbarium specimens at the DNA level, developing a molecular biogeographical population survey in relation to vigor (*in situ* and *ex situ*) of the CD genome forms, making use of the *Oryza* Map Alignment Project with several hundred thousands BAC end sequences available [32], and artificially reconstituting the CD genome through interspecific hybrization.

## Supporting Information

Table S1 Specimens of the ‘*O. sativa* complex’, and *Rhynchoryza subulata* collected in the Americas in the 18^th^ and 19^th^ centuries, and two *O. officinalis* from Java mentioned in the text, as observed from herbariums.
(0.07 MB DOC)
