# Supplementary material for: Human-Mediated Emergence as a Weed and Invasive Radiation in the Wild of the CD Genome Allotetraploid Rice Species (Oryza, Poaceae) in the Neotropics
Source: PLoS One. 2008 Jul 2;3(7):e2613. doi: 10.1371/journal.pone.0002613 (PMC2434201; doi:10.1371/journal.pone.0002613)
Supplement: Table S1 — Specimens of the ‘O. sativa complex’, and Rhynchoryza subulata collected in the Americas in the 18th and 19th centuries, and two O. officinalis from Java mentioned in the text, as observed from herbariums. (0.07 MB DOC) [file pone.0002613.s001.doc]

**Table S1.** Specimens of the ‘*O. sativa* complex’, and *Rhynchoryza subulata* collected in the Americas in the 18^th^ and 19^th^ centuries, and two *O. officinalis* from Java mentioned in the text, as observed from herbariums.

| **Species** | **Country ^a^** | **Locality** | **Collector’s  name** | **Date of  collection ^a^** | **Herbarium ^b^** | **Name of image (images available upon request)** |
| --- | --- | --- | --- | --- | --- | --- |
| *O. barthii* | Brazil | Pará, Rio Negro, Mato Grosso | C.F.P. Martius | *ca* 1818 | M | M2 |
| *O. glaberrima* | Antilles? | Possibly Cuba | V.T. Gibollet | 1847 | G | G12 |
| *O. glaberrima* | Cuba | – | – | *ca* 1877 | BM | BM8 |
| *O. sativa* | USA | Carolina | – | 1783 | MPU | MPU4a, MPU4b |
| *O. sativa* | Puerto Rico | – | A.P. Ledru | *ca* 1797 | G | G2 |
| *O. sativa* | S^t^ Thomas | – | Riedley | – | MPU | MPU6 |
| *O. sativa / O. glumaepatula* | Brazil | Piaui | C.F.P. Martius | *ca* 1818 | M | M1 |
| *O. sativa* | Mexico | Cultivated | C.J.W. Schiede & F. Deppe | 1828 | BM | BM4 |
| *O. sativa* | USA | Louisiana, Covington' | Drummond | 1832 | BM | BM9 |
| *O. sativa* | Brazil | Rio San Francisco. Cultivated. | G. Gardner | 1838 | BM | BM1, BM2 |
| *O. sativa / O. glumaepatula* | Brazil | Santarem, Pará | R. Spruce | 1850 | CGE | CGE1 |
| *O. sativa* | Panama | – | S. Hayes | *ca* 1865 | BM | BM3 |
| *O. sativa* | Brazil | Lagoa Santa | J.E.B. Warming | 1864 | C | C2, C3 |
| *O. glumaepatula* | Brazil | Mato Grosso, and Pará | B. Luschnath | 1828 | LE | LE5 |
| *O. glumaepatula* | Surinam | – | C.F.F. Hochst | 1842 | P | P14, P16, P17 (type) |
| *O. glumaepatula* | Brazil | Valle Broco | B. Luschnath | 1845 | LE | LE6 |
| *O. glumaepatula* | Brazil | Mato Grosso: Rio Guaporé | – | – | LE | LE7 |
| *O. glumaepatula* | Brazil | Pará: Santarem, Rio Trombetas | R. Spruce | 1850 | C, G, P | G11, C1, P18 |
| *R. subulata* | Brazil | South of Brazil | Schlechtendal | 1827 |  | LE8 |
| *R. subulata* | Paraguay | Rio Tebicuari | B. Balansa | 1875 | S | S3 |
| *O. officinalis* | Indonesia | Java | P. Commerson | 1768 | MPU | MPU7 |
| *O. officinalis* | Indonesia | Java | P. Commerson | 1768 | P | P13 |

^a^ As indicated on the label, or as deduced from the locality and/or literatures and explorer’s reports.

^b^ Herbarium acronyms, according to Holmgren *et al.* [39].
